# ^177^Lu-Prostate-Specific Membrane Antigen Ligand After ^223^Ra Treatment in Men with Bone-Metastatic Castration-Resistant Prostate Cancer: Real-World Clinical Experience

**DOI:** 10.2967/jnumed.121.262240

**Published:** 2022-03

**Authors:** Oliver Sartor, Christian la Fougère, Markus Essler, Samer Ezziddin, Gero Kramer, Jörg Ellinger, Luke Nordquist, John Sylvester, Giovanni Paganelli, Avivit Peer, Martin Bögemann, Jeffrey Meltzer, Per Sandström, Frank Verholen, Daniel Y. Song

**Affiliations:** 1 Tulane Cancer Center, Tulane University School of Medicine, New Orleans, Louisiana;; 2 Department of Nuclear Medicine and Clinical Molecular Imaging, University Hospital Tübingen, Tübingen, Germany;; 3 Clinic and Polyclinic for Nuclear Medicine, University Hospital Bonn, Bonn, Germany;; 4 Department of Nuclear Medicine, Saarland University, Homburg, Germany;; 5 Urology Clinic, Medical University of Vienna, Vienna, Austria;; 6 Clinic for Urology and Pediatric Urology, University Hospital Bonn, Bonn, Germany;; 7 Guam Research Network LLC, Omaha, Nebraska;; 8 21st Century Oncology–Sarasota, Lakewood Ranch, Florida;; 9 Scientific Institute of Romagna for the Study and Treatment of Tumors, UOC Nuclear Medicine, Meldola, Italy;; 10 Rambam MC, Haifa, Israel;; 11 Department of Urology, University Hospital Münster, Münster, Germany;; 12 Bayer HealthCare, Whippany, New Jersey;; 13 Bayer Consumer Care, Basel, Switzerland;; 14 Radiation Oncology, Johns Hopkins University, Baltimore, Maryland

**Keywords:** ^177^Lu-prostate-specific membrane antigen, metastatic castration-resistant prostate cancer, ^223^Ra, real-world evidence, treatment sequence

## Abstract

We analyzed real-world clinical outcomes of sequential α-/β-emitter therapy for metastatic castration-resistant prostate cancer (mCRPC). **Methods:** We assessed safety and overall survival in 26 patients who received ^177^Lu-prostate-specific membrane antigen ligand (^177^Lu-PSMA) after ^223^Ra in the ongoing noninterventional REASSURE study (^223^Ra α-Emitter Agent in Nonintervention Safety Study in mCRPC Population for Long-Term Evaluation; NCT02141438). **Results:** Patients received ^223^Ra for a median of 6 injections and subsequent ^177^Lu-PSMA for a median of 3.5 mo (≥ the fourth therapy in 69%). The median time between ^223^Ra and ^177^Lu-PSMA treatment was 8 mo (range, 1–31 mo). Grade 3 hematologic events occurred in 9 of 26 patients (during or after ^177^Lu-PSMA treatment in 5/9 patients; 8/9 patients had also received docetaxel). Median overall survival was 28.0 mo from the ^223^Ra start and 13.2 mo from the ^177^Lu-PSMA start. **Conclusion:** Although the small sample size precludes definitive conclusions, these preliminary data, especially the ^177^Lu-PSMA treatment duration, suggest that the use of ^177^Lu-PSMA after ^223^Ra is feasible in this real-world setting.

## Visual Abstract

**Figure:**
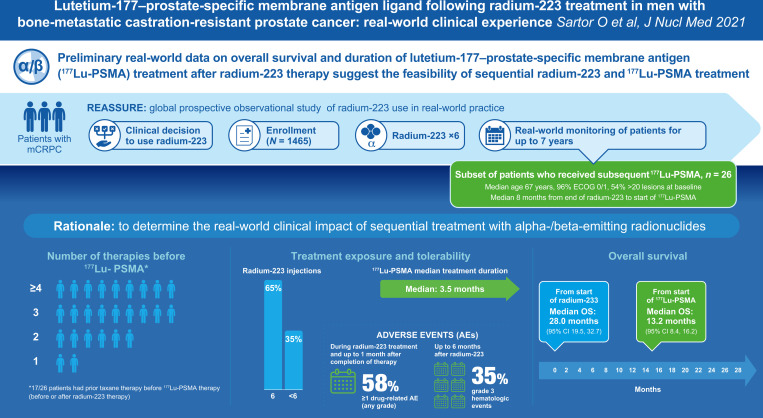

The α-emitter ^223^Ra demonstrated significantly prolonged overall survival and a favorable safety profile versus placebo in men with metastatic castration-resistant prostate cancer (mCRPC) in the phase 3 ALSYMPCA trial (*1*). ^177^Lu-prostate-specific membrane antigen ligand (^177^Lu-PSMA) is an investigational β-emitting radioligand with accumulating evidence of clinical efficacy and acceptable toxicity in men with advanced-stage mCRPC (*2*–*5*).

Early experience in patients who have received both ^223^Ra and ^177^Lu-PSMA indicates tolerable safety and therapeutic response with this sequence (*6*–*8*). We sought to add to the evidence base on sequential α-/β-emitting therapy, using data from participants in an ongoing global, prospective, observational study of ^223^Ra who received subsequent ^177^Lu-PSMA.

## MATERIALS AND METHODS

Patients with mCRPC involving bone and who were scheduled to receive ^223^Ra in clinical practice were included in REASSURE (^223^Ra α-Emitter Agent in Nonintervention Safety Study in mCRPC Population for Long-Term Evaluation; NCT02141438). Primary outcomes included short-term and long-term safety. Methods and results from a previous interim analysis have been reported (*9*). This paper is based on the second prespecified interim analysis (data cutoff, March 20, 2019).

Disease characteristics, adverse events after ^223^Ra treatment, and overall survival are described for patients who received the experimental drug ^177^Lu-PSMA in compassionate-use or investigational settings after ^223^Ra. Treatment-emergent serious adverse events and drug-related adverse events were recorded during ^223^Ra treatment or up to 30 d after the last ^223^Ra dose. Grade 3 or 4 hematologic adverse events were systematically collected up to 6 mo after ^223^Ra; neutropenic fever or hemorrhage were recorded in patients with subsequent chemotherapy up to 6 mo after the last dose of chemotherapy. Drug-related serious adverse events continued to be recorded until the end of follow-up (maximum, 7 y). Adverse events during and after ^177^Lu-PSMA therapy were not systematically recorded unless they met the above criteria.

The study conduct complied with the requirements of the European Medicines Agency, the U.S. Food and Drug Administration, applicable local laws and regulations, and International Conference on Harmonization good-clinical-practice guidance. Participants provided written informed consent, and ethics committee or institutional review board approvals were obtained according to local laws in participating countries.

## RESULTS

Twenty-six patients in the United States, Germany, Austria, Italy, and Israel received ^177^Lu-PSMA after ^223^Ra. Their median age was 67 y, 96% (25/26) had an Eastern Cooperative Oncology Group performance status of 0 or 1, and 54% (13/24 with baseline scans) had more than 20 lesions at baseline (Table 1).

**TABLE 1:** Baseline Disease Characteristics

Time point	Characteristic	Finding	Data
Initial diagnosis	Gleason score	≤6	3 (12)
		7	9 (35)
		8–10	12 (46)
		Unknown	2 (8)
	Stage (American Joint Committee on Cancer criteria)	I	5 (19)
		IIB	1 (4)
		III	3 (12)
		IV	13 (50)
		Missing	4 (15)
Start of ^223^Ra therapy	Time from diagnosis of mCRPC (mo)		20 (6–48)
	Time from diagnosis of bone metastases (mo)		23 (3–40)
	Extent of disease*	<6 lesions	2 (8)
		6–20 lesions	7 (29)
		>20 lesions	11 (46)
		Superscan	2 (8)
		Missing	2 (8)
	Primary tumor status	Unresected	11 (42)
		Resected, status of residual tumor unknown	3 (12)
		R0 complete resection, all margins histologically negative	6 (23)
		R1 incomplete resection, microscopic margin involvement	5 (19)
		Missing	1 (4)
	Laboratory values	Prostate-specific antigen (ng/mL) (*n* = 21)	127 (8–1,319)
		Alkaline phosphatase (U/L) (*n* = 20)	147 (45–769)
		Lactate dehydrogenase (U/L) (*n* = 14)	228 (112–393)
		Hemoglobin (g/dL) (*n* = 23)	13 (9–15)

*Baseline scan data available for 24/26 patients.

Qualitative data are number and percentage (*n* = 26 unless indicated otherwise); continuous data are median and range.

Before starting ^223^Ra, 85% of patients (22/26) received at least 1 life-prolonging systemic anticancer therapy (Supplemental Fig. 1; supplemental materials are available at http://jnm.snmjournals.org), including androgen receptor–targeted therapy (enzalutamide and/or abiraterone acetate) in 65% (17/26) and docetaxel in 42% (11/26).

**FIGURE 1.**
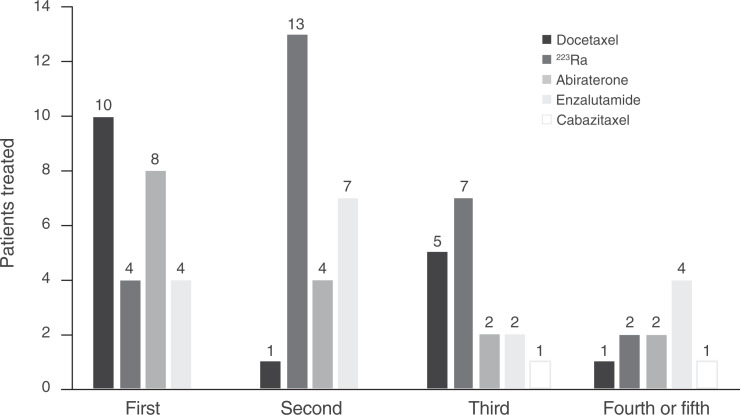
Anticancer therapies administered before ^177^Lu–PSMA. All patients received ^223^Ra.

Before starting ^177^Lu-PSMA, 92% of patients (24/26) had received at least 2 life-prolonging therapies, 69% (18/26) had received at least 3 therapies, 8% (2/26) had received only ^223^Ra, 65% (17/26) had received prior docetaxel, 8% (2/26) had also received cabazitaxel between ^223^Ra and ^177^Lu-PSMA treatment, and 50% (13/26) had received no other life-prolonging treatment between ^223^Ra and ^177^Lu-PSMA (Fig. 1; Supplemental Fig. 1).

**FIGURE 2.**
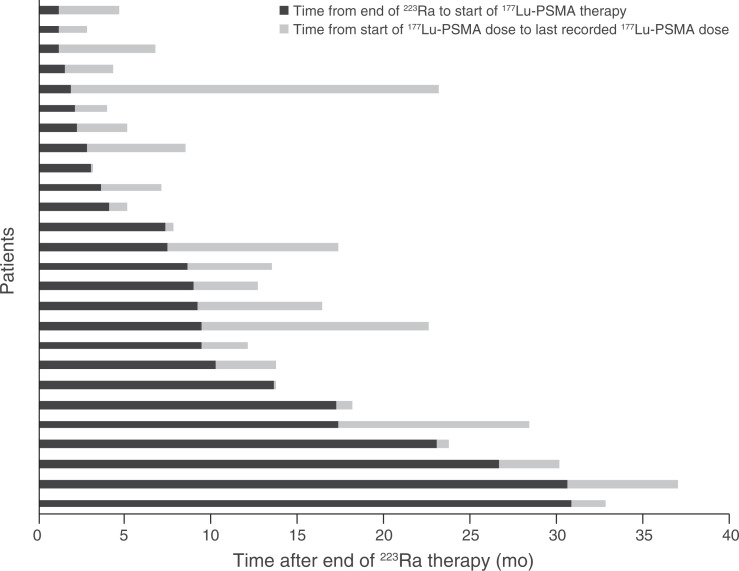
Time since end of ^223^Ra to start of ^177^Lu-PSMA ligand and duration of ^177^Lu-PSMA therapy.

The median number of ^223^Ra injections was 6 (range, 1–6); 17 of 26 patients (65%) received 6 injections. The median time from the end of ^223^Ra to the start of ^177^Lu-PSMA treatment was 8 mo (range, 1–31 mo; Fig. 2). The median duration of ^177^Lu-PSMA treatment was 3.5 mo (range, 0.5–21.2 mo; Fig. 2).

Fifteen patients (58%) experienced treatment-emergent drug-related adverse events during ^223^Ra treatment (Table 2). Nine patients (35%) had grade 3 hematologic toxicities (Table 3); 8 of 9 patients had previously received docetaxel, before (*n* = 5) or after (*n* = 3) ^223^Ra therapy, and 2 of 9 patients had also received cabazitaxel after ^223^Ra. The hematologic toxicities developed during or after ^177^Lu-PSMA treatment in 5 patients (6 events). No grade 4 hematologic events were recorded.

**TABLE 2:** Adverse Events During and After ^223^Ra Treatment

Adverse event	Incidence (*n* = 26)
Drug-related	
Treatment-emergent*	15 (58%)
Serious^†^	3 (12%)
Bone-associated events	6 (23%)
Fractures	2 (8%)
Bone disorders^‡^	4 (15%)

*During ^223^Ra therapy and up to 30 d after last ^223^Ra dose.

^†^During ^223^Ra therapy and up to 7 y after last ^223^Ra dose.

^‡^Excluding congenital disorders and fractures, according to *Medical Dictionary for Regulatory Activities,* version 21.1 (https://www.meddra.org/).

Qualitative data are number and percentage.

**TABLE 3:** Grade 3 Hematologic Adverse Events After Start of ^223^Ra Therapy*

	Incidence (*n* = 26)		
Patients with events^†^	Overall	Starting before ^177^Lu-PSMA treatment	Starting during or after ^177^Lu-PSMA treatment^‡^
Any	9 (35%)	5 (19%)	5 (19%)
Leukopenia	0	0	0
Neutropenia	0	0	0
Pancytopenia	1 (4%)	0	1 (4%)
Thrombocytopenia	3 (12%)	2 (8%)	1 (4%)
Anemia	6 (23%)	3 (12%)	4 (15%)

*No grade ≥4 events were recorded.

^†^Patients may have had >1 event at different times; these patients are counted only once in “Any” row and “Overall” column.

^‡^Grade 3/4 hematologic toxicity data were systematically recorded only up to 6 mo after completion of ^223^Ra therapy; data are therefore not consistently available for patients who received ^177^Lu-PSMA after this window.

Qualitative data are number and percentage.

Median overall survival was 28.0 mo (95% CI, 19.5–32.7 mo) from the start of ^223^Ra therapy and 13.2 mo (95% CI, 8.4–16.2 mo) from the start of ^177^Lu-PSMA therapy.

## DISCUSSION

Although ^177^Lu-PSMA is not yet approved for patients with mCRPC, patients are increasingly receiving this investigational treatment in clinical trials or compassionate-use programs. Most patients receive ^177^Lu-PSMA after multiple prior systemic anticancer therapies, including ^223^Ra in some cases, as recorded in the REASSURE study. This subgroup analysis of REASSURE, which reflects real-world clinical practice, adds to the evidence for the feasibility of sequential ^223^Ra and ^177^Lu-PSMA treatment, with a median overall survival of more than 1 y from the start of ^177^Lu-PSMA therapy. Only 3 patients had serious adverse events related to ^223^Ra, and the reported (albeit incompletely) incidence of grade 3 hematologic events was acceptable, mostly consisting of anemia, which may be partially explained by increasing disease burden. Furthermore, the treatment duration for ^177^Lu-PSMA (median, 3.5 mo) indicates that several patients were able to receive multiple cycles, even though most patients had received at least 3 prior life-prolonging therapies, including taxane chemotherapy.

The 13-mo median overall survival in our analysis is consistent with a retrospective multicenter study in which median overall survival from the start of ^177^Lu-PSMA therapy was around 11 mo in 85 patients with prior ^223^Ra (*7*) and 16.4 mo in patients with 6–20 bone lesions treated with ^223^Ra and ^177^Lu-PSMA (*10*). In another analysis, rates of grade 3 hematologic toxicity were low in patients with or without prior ^223^Ra therapy (anemia, 1/20 [5%] vs. 3/29 [10%]; thrombocytopenia, 1/20 [5%] vs. 2/29 [7%]) (*6*), a result that again supports our findings, although we did not systematically assess hematologic toxicity in all patients during ^177^Lu-PSMA treatment—a limitation of our study.

Additional limitations are the small sample size, reflecting the experimental status of ^177^Lu-PSMA, and the lack of a randomized control group. Because ^177^Lu-PSMA is still an investigational agent, treatment was likely undertaken in academic settings (e.g., university hospital cancer centers); it is therefore unknown whether the findings can be extrapolated to real-world community settings. The treatment duration and overall survival after ^177^Lu-PSMA initiation indicate that its use after ^223^Ra in heavily pretreated mCRPC patients is feasible, but interpretation is hindered by lack of a comparator arm, and possibly only the fittest patients were selected for ^177^Lu-PSMA treatment. Nevertheless, this interim analysis of an ongoing real-world study provides clinically meaningful evidence in patients with mCRPC who successfully received sequential α-/β-emitting treatments.

## CONCLUSION

In this real-world population of heavily pretreated patients with mCRPC, a treatment sequence of targeted α-therapy with ^223^Ra followed by the β-emitter ^177^Lu-PSMA seemed feasible, based on the duration of ^177^Lu-PSMA therapy, although definitive conclusions cannot be drawn.

## DISCLOSURE

Oliver Sartor reports grants or fees from Amgen, Bayer, Sanofi, AstraZeneca, Dendreon, Constellation Pharmaceuticals, Advanced Accelerator Applications, Endocyte, Pfizer, Bristol Myers Squibb, Bavarian Nordic, EMD Serono, Astellas Pharma, Progenics, Blue Earth Diagnostics, Merck, Invitae, Astellas, Endocyte, Myovant Sciences, Myriad Genetics, Novartis, Clarity Pharmaceuticals, Fusion Pharmaceuticals, Isotopen Technologien, Janssen, Noxopharm, Clovis Oncology, Taiho, Noria Therapeutics, Point Biopharma, TeneoBio, Telix Pharmaceuticals, and Theragnostics. Christian la Fougère serves as a consultant/adviser for Bayer and Sanofi-Aventis. Markus Essler reports research funding from Novartis; is a consultant/adviser for Bayer, Novartis, and Ipsen; and receives travel expenses from Ipsen and Sirtex. Samer Ezziddin reports travel expenses from Ipsen. Jörg Ellinger serves as a consultant for Bayer. John Sylvester reports employment at 21^st^ Century Oncology; research funding from Prostatak (via 21^st^ Century Oncology); stock in Augmenix; patents, royalties, or other intellectual properties with Myriad; and honoraria from Decipher and Theragenics. John Sylvester also serves as a consultant/adviser for, receives travel expenses from, and is on the speakers’ bureau for Theragenics. Avivit Peer serves as a consultant/adviser for Pfizer, BMS, Roche, Eisai, MSD, Janssen, Astellas, Novartis, Medison, AstraZeneca, and Bayer. Jeffrey Meltzer, Per Sandström, and Frank Verholen are employees of Bayer. Daniel Song reports research funding from Bayer, Advantagene, Bristol Myers Squibb, and BioProtect and serves as a consultant/adviser for BioProtect. This work was supported by Bayer Healthcare Pharmaceuticals Inc., Whippany, NJ, USA. No other potential conflict of interest relevant to this article was reported.

## Notes

KEY POINTS **QUESTION:** Is it feasible to treat men with mCRPC with sequential α- and β-emitting therapies? **PERTINENT FINDINGS:** Subgroup analysis of a global observational study of ^223^Ra therapy indicated a low rate of serious adverse events and hematologic toxicities in patients who also received ^177^Lu-PSMA, and many patients were able to receive multiple doses of ^177^Lu-PSMA (a marker of tolerability). This sequence provides overall survival of more than 2 y from the initiation of ^223^Ra and more than 1 y from the initiation of ^177^Lu-PMSA, even in heavily pretreated patients. **IMPLICATIONS FOR PATIENT CARE:** Sequential use of α- and β-emitters appears to be feasible in selected patients, on the basis of the known safety profile of ^223^Ra and the duration of subsequent ^177^Lu-PSMA; this sequence warrants further investigation.
